# Supplementary material for: Genomic epidemiology of the early stages of the SARS-CoV-2 outbreak in Russia
Source: Nat Commun. 2021 Jan 28;12:649. doi: 10.1038/s41467-020-20880-z (PMC7844267; doi:10.1038/s41467-020-20880-z)
Supplement: Supplementary file 1 — Supplementary Information [file 41467_2020_20880_MOESM1_ESM.pdf]

Supplementary Information

Supplementary Note

To estimate the number of introduction events, we used the following procedure. When a Russian lineage or singleton had no Russian sequences in their stem (e.g., Figs. 5c, 5d, 6a), we assumed that they originated from distinct introductions. There were 3 such Russian transmission lineages (lineages 2, 3 and 9) which together included 8 sequences; and 33 such singletons (Supplementary Figs. 2-3), for a total of 36 introduction events.

Additionally, some of the Russian lineages descended from internal nodes with a mix of Russian and non-Russian sequences (e.g., Fig. 5a-b). Similarly, in a fraction of cases, a Russian singleton descended from an internal node with multiple sequences corresponding to it, such that some of them were Russian. These cases are referred to as Russian stem-derived transmission lineages and Russian stem-derived singletons, respectively. In these cases, whether the origin of the lineage or singleton corresponded to an introduction event could not be established unambiguously. Finally, each stem cluster could also originate from any number of introductions, ranging between 1 (if all transmissions within it were domestic) and the number of sequences in the cluster (if each sequence was introduced independently) (Fig. 4).

To address this, we used the following statistical procedure. We used the fact that for a fraction of samples, direct travel data were available (Supplementary Data 4): we had information on travel abroad or absence of travel history of the sampled individuals. We assumed that these data are reflective of the fraction of sequences in the corresponding category (stem-derived transmission lineages, stem-derived singletons or stem clusters) that were introduced, and that this fraction is reflective of the entire category of samples. For transmission lineages, we assumed that if at least some individuals travelled abroad, this lineage was introduced; and if some of the individuals had documented absence of travel (but none had travelled abroad), this lineage was not introduced. Therefore, for each category , we estimated the number of introductions as , where is the number of sequenced lineages, or sequenced samples in a non-lineage category; is the number of samples among them with documented travel history; and is the number of samples among them with documented absence of travel history (Supplementary Table 1).

Using this procedure, we estimate that sequences among these three categories result from additional ~3 introductions yielding transmission lineages (three of the lineages 1, 4, 5, 6, 7 and 8 with Russian sequences at the ancestral node); ~6 introductions yielding some of the 40 singletons with Russian sequences at ancestral nodes; and ~22 introductions yielding Russian sequences in stem clusters. Therefore, we estimate the total number of introductions yielding the sampled diversity in Russia as 36+31=67. This number provides a conservative estimate for the number of introductions. It is likely an underestimate; e.g., if many of the singletons are actually reflective of unsampled Russian transmission lineages, and the index case of these

lineages was never sampled, singleton individuals without travel history may still reflect distinct introductions.

Supplementary Figures

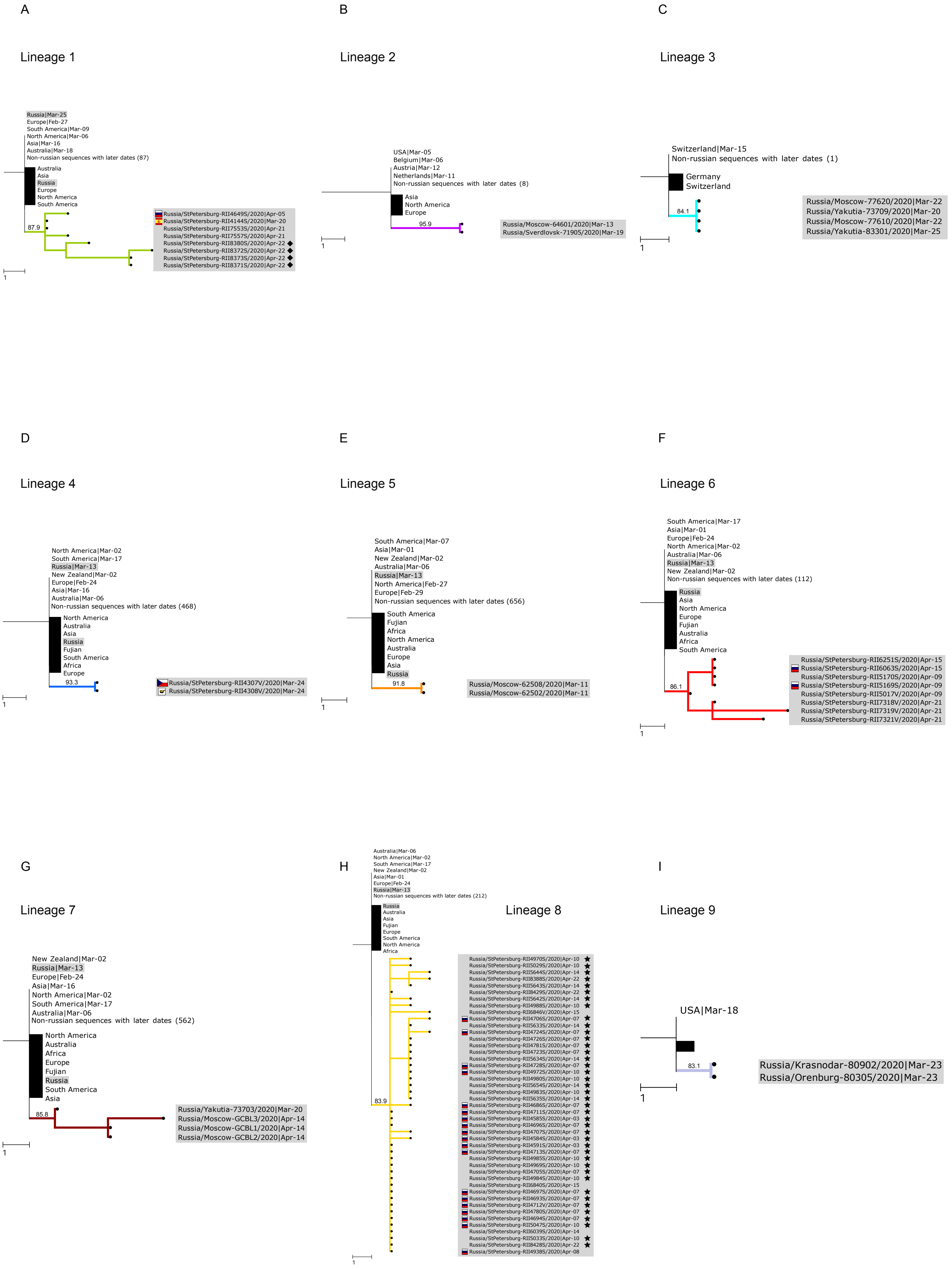

A
Lineage 1
Russia|Mar-25
Europe|Feb-27
South America|Mar-09
North America|Mar-06
Asia|Mar-16
Australia|Mar-18
Non-russian sequences with later dates (87)
Australia
Asia
Russia
Europe
North America
South America
87.9
Russia/StPetersburg-RII4649S/2020|Apr-05
Russia/StPetersburg-RII4144S/2020|Mar-20
Russia/StPetersburg-RII7553S/2020|Apr-21
Russia/StPetersburg-RII7557S/2020|Apr-21
Russia/StPetersburg-RII8380S/2020|Apr-22
Russia/StPetersburg-RII8372S/2020|Apr-22
Russia/StPetersburg-RII8373S/2020|Apr-22
Russia/StPetersburg-RII8371S/2020|Apr-22
1
B
Lineage 2
USA|Mar-05
Belgium|Mar-06
Austria|Mar-12
Netherlands|Mar-11
Non-russian sequences with later dates (8)
Asia
North America
Europe
95.9
Russia/Moscow-64601/2020|Mar-13
Russia/Sverdlovsk-71905/2020|Mar-19
1
C
Lineage 3
Switzerland|Mar-15
Non-russian sequences with later dates (1)
Germany
Switzerland
84.1
Russia/Moscow-77620/2020|Mar-22
Russia/Yakutia-73709/2020|Mar-20
Russia/Moscow-77610/2020|Mar-22
Russia/Yakutia-83301/2020|Mar-25
1
D
Lineage 4
North America|Mar-02
South America|Mar-17
Russia|Mar-13
New Zealand|Mar-02
Europe|Feb-24
Asia|Mar-16
Australia|Mar-06
Non-russian sequences with later dates (468)
North America
Australia
Asia
Russia
Fujian
South America
Africa
Europe
93.3
Russia/StPetersburg-RII4307V/2020|Mar-24
Russia/StPetersburg-RII4308V/2020|Mar-24
1
E
Lineage 5
South America|Mar-07
Asia|Mar-01
New Zealand|Mar-02
Australia|Mar-06
Russia|Mar-13
North America|Feb-27
Europe|Feb-29
Non-russian sequences with later dates (656)
South America
Fujian
Africa
North America
Australia
Europe
Asia
Russia
91.8
Russia/Moscow-62508/2020|Mar-11
Russia/Moscow-62502/2020|Mar-11
1
F
Lineage 6
South America|Mar-17
Asia|Mar-01
Europe|Feb-24
North America|Mar-02
Australia|Mar-06
Russia|Mar-13
New Zealand|Mar-02
Non-russian sequences with later dates (112)
Russia
Asia
North America
Europe
Fujian
Australia
Africa
South America
86.1
Russia/StPetersburg-RII6251S/2020|Apr-15
Russia/StPetersburg-RII6063S/2020|Apr-15
Russia/StPetersburg-RII5170S/2020|Apr-09
Russia/StPetersburg-RII5169S/2020|Apr-09
Russia/StPetersburg-RII5017V/2020|Apr-09
Russia/StPetersburg-RII7318V/2020|Apr-21
Russia/StPetersburg-RII7319V/2020|Apr-21
Russia/StPetersburg-RII7321V/2020|Apr-21
1
G
Lineage 7
New Zealand|Mar-02
Russia|Mar-13
Europe|Feb-24
Asia|Mar-16
North America|Mar-02
South America|Mar-17
Australia|Mar-06
Non-russian sequences with later dates (562)
North America
Australia
Africa
Europe
Fujian
Russia
South America
Asia
85.8
Russia/Yakutia-73703/2020|Mar-20
Russia/Moscow-GCBL3/2020|Apr-14
Russia/Moscow-GCBL1/2020|Apr-14
Russia/Moscow-GCBL2/2020|Apr-14
1
H
Lineage 8
Australia|Mar-06
North America|Mar-02
South America|Mar-17
New Zealand|Mar-02
Asia|Mar-01
Europe|Feb-24
Russia|Mar-13
Non-russian sequences with later dates (212)
Russia
Australia
Asia
Fujian
Europe
South America
North America
Africa
83.9
Russia/StPetersburg-RII4970S/2020|Apr-10
Russia/StPetersburg-RII5029S/2020|Apr-10
Russia/StPetersburg-RII5644S/2020|Apr-14
Russia/StPetersburg-RII8388S/2020|Apr-22
Russia/StPetersburg-RII5643S/2020|Apr-14
Russia/StPetersburg-RII8429S/2020|Apr-22
Russia/StPetersburg-RII5642S/2020|Apr-14
Russia/StPetersburg-RII4988S/2020|Apr-10
Russia/StPetersburg-RII6846V/2020|Apr-15
Russia/StPetersburg-RII4706S/2020|Apr-07
Russia/StPetersburg-RII5633S/2020|Apr-14
Russia/StPetersburg-RII4724S/2020|Apr-07
Russia/StPetersburg-RII4726S/2020|Apr-07
Russia/StPetersburg-RII4781S/2020|Apr-07
Russia/StPetersburg-RII4723S/2020|Apr-07
Russia/StPetersburg-RII5634S/2020|Apr-14
Russia/StPetersburg-RII4728S/2020|Apr-07
Russia/StPetersburg-RII4972S/2020|Apr-10
Russia/StPetersburg-RII4980S/2020|Apr-10
Russia/StPetersburg-RII5654S/2020|Apr-14
Russia/StPetersburg-RII4983S/2020|Apr-10
Russia/StPetersburg-RII5635S/2020|Apr-14
Russia/StPetersburg-RII4686S/2020|Apr-07
Russia/StPetersburg-RII4711S/2020|Apr-07
Russia/StPetersburg-RII4585S/2020|Apr-03
Russia/StPetersburg-RII4696S/2020|Apr-07
Russia/StPetersburg-RII4707S/2020|Apr-07
Russia/StPetersburg-RII4584S/2020|Apr-03
Russia/StPetersburg-RII4591S/2020|Apr-03
Russia/StPetersburg-RII4713S/2020|Apr-07
Russia/StPetersburg-RII4985S/2020|Apr-10
Russia/StPetersburg-RII4969S/2020|Apr-10
Russia/StPetersburg-RII4705S/2020|Apr-07
Russia/StPetersburg-RII4984S/2020|Apr-10
Russia/StPetersburg-RII6840S/2020|Apr-15
Russia/StPetersburg-RII4697S/2020|Apr-07
Russia/StPetersburg-RII4693S/2020|Apr-07
Russia/StPetersburg-RII4712V/2020|Apr-07
Russia/StPetersburg-RII4780S/2020|Apr-07
Russia/StPetersburg-RII4694S/2020|Apr-07
Russia/StPetersburg-RII5047S/2020|Apr-10
Russia/StPetersburg-RII6039S/2020|Apr-14
Russia/StPetersburg-RII5033S/2020|Apr-10
Russia/StPetersburg-RII8428S/2020|Apr-22
Russia/StPetersburg-RII4938S/2020|Apr-08
1
I
Lineage 9
USA|Mar-18
83.1
Russia/Krasnodar-80902/2020|Mar-23
Russia/Orenburg-80305/2020|Mar-23
1

Supplementary Figure 1. Russian transmission lineages and stem-derived transmission lineages. Each panel (A-I) shows an independent transmission lineage. Lineage colors are as in Figs. 2-3 and 5-6. Notation as in Fig. 5. Stars and diamonds in (A) and (I) indicate samples associated with the Vreden hospital (see Fig. 9).

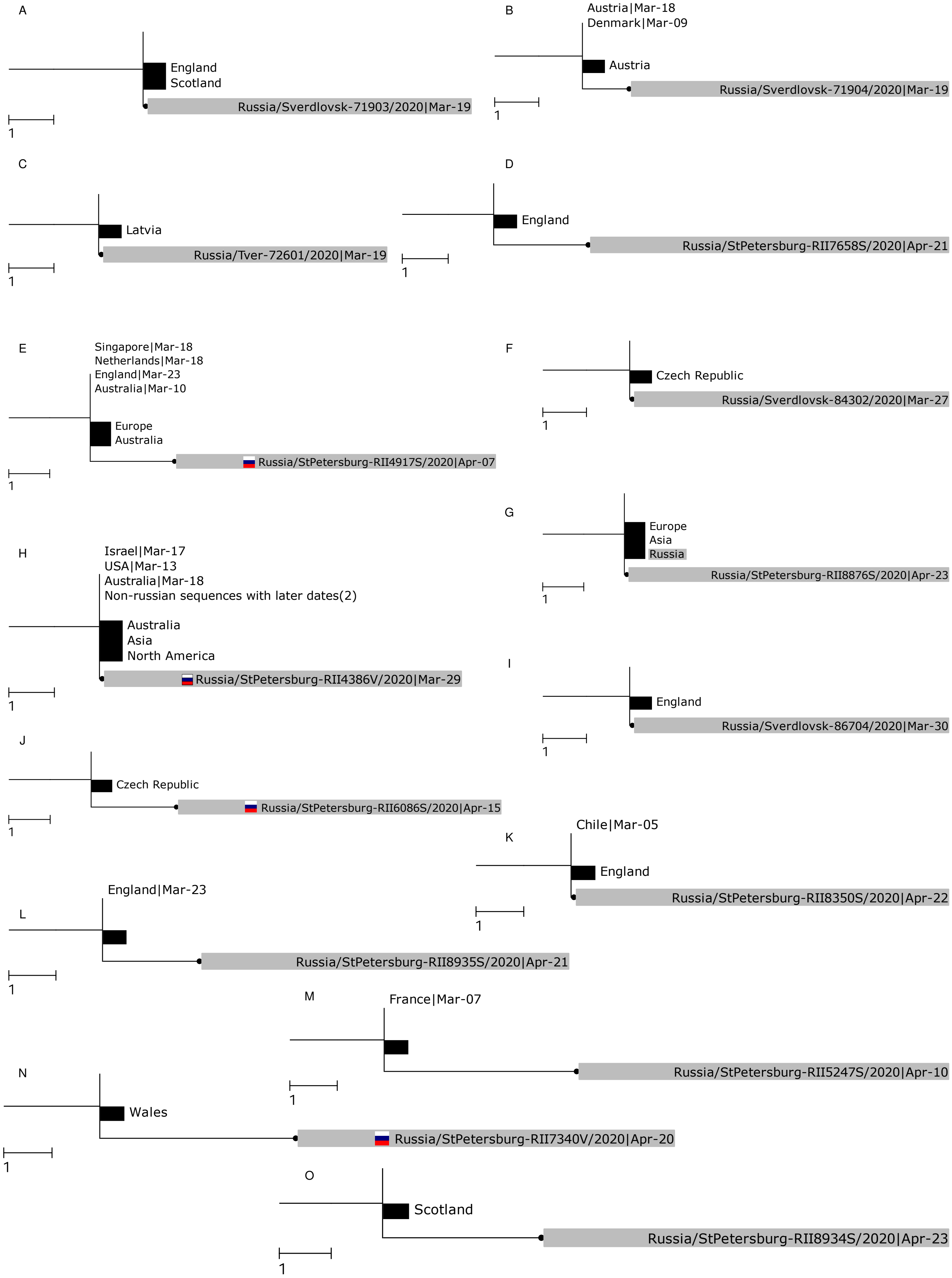

A
England
Scotland
Russia/Sverdlovsk-71903/2020|Mar-19
1
B
Austria|Mar-18
Denmark|Mar-09
Austria
Russia/Sverdlovsk-71904/2020|Mar-19
1
C
Latvia
Russia/Tver-72601/2020|Mar-19
1
D
England
Russia/StPetersburg-RII7658S/2020|Apr-21
1
E
Singapore|Mar-18
Netherlands|Mar-18
England|Mar-23
Australia|Mar-10
Europe
Australia
Russia/StPetersburg-RII4917S/2020|Apr-07
1
F
Czech Republic
Russia/Sverdlovsk-84302/2020|Mar-27
1
G
Europe
Asia
Russia
Russia/StPetersburg-RII8876S/2020|Apr-23
1
H
Israel|Mar-17
USA|Mar-13
Australia|Mar-18
Non-russian sequences with later dates(2)
Australia
Asia
North America
Russia/StPetersburg-RII4386V/2020|Mar-29
1
I
England
Russia/Sverdlovsk-86704/2020|Mar-30
1
J
Czech Republic
Russia/StPetersburg-RII6086S/2020|Apr-15
1
K
Chile|Mar-05
England
Russia/StPetersburg-RII8350S/2020|Apr-22
1
L
England|Mar-23
Russia/StPetersburg-RII8935S/2020|Apr-21
1
M
France|Mar-07
Russia/StPetersburg-RII5247S/2020|Apr-10
1
N
Wales
Russia/StPetersburg-RII7340V/2020|Apr-20
1
O
Scotland
Russia/StPetersburg-RII8934S/2020|Apr-23
1

Supplementary Figure 2. Russian singletons (continued on Supplementary Figure 3). Each panel (A-O) shows an individual Russian singleton. Notation as in Figs. 5-6.

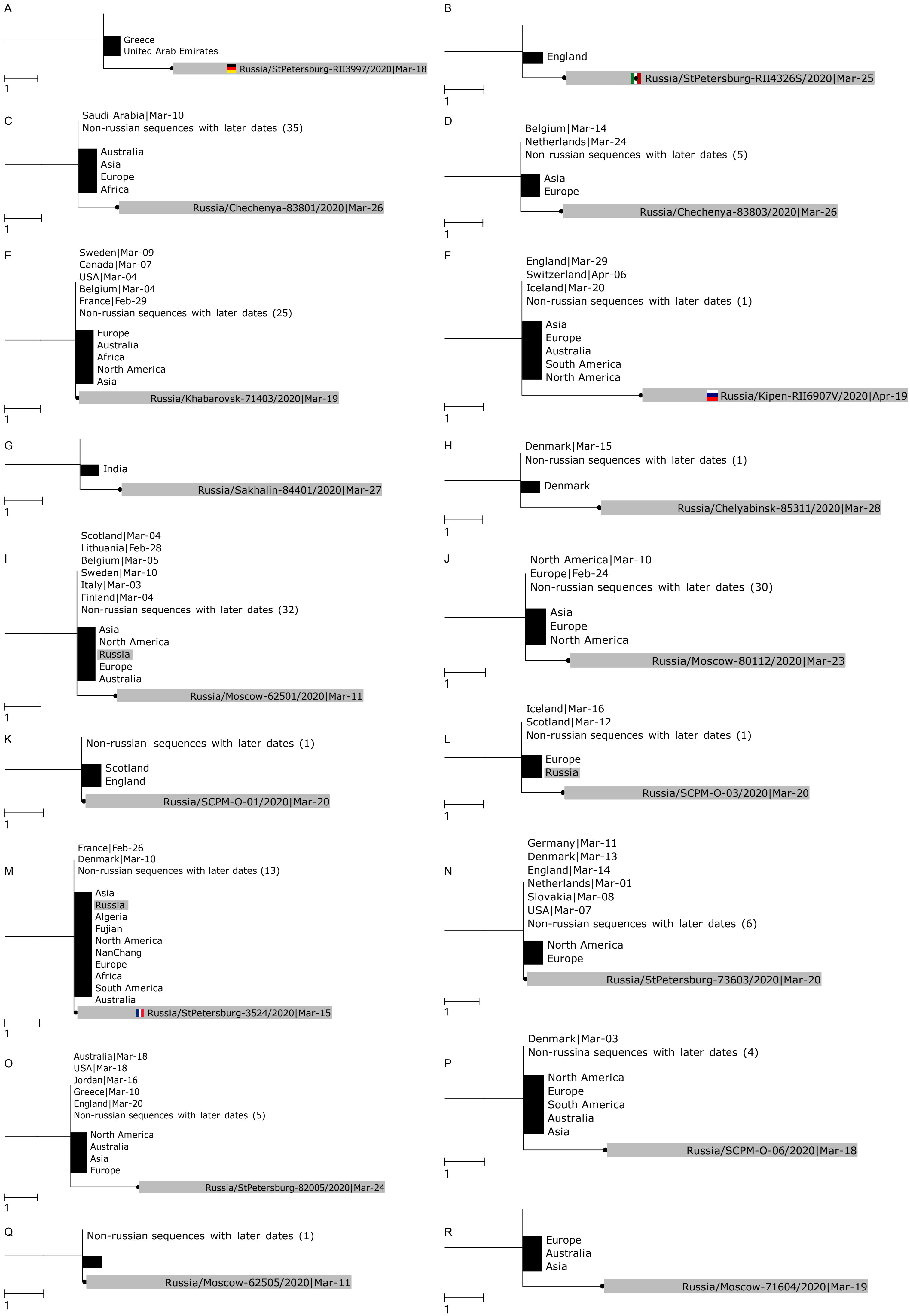
A
Greece
United Arab Emirates
Russia/StPetersburg-RII3997/2020|Mar-18
1
B
England
Russia/StPetersburg-RII4326S/2020|Mar-25
1
C
Saudi Arabia|Mar-10
Non-russian sequences with later dates (35)
Australia
Asia
Europe
Africa
Russia/Chechenya-83801/2020|Mar-26
1
D
Belgium|Mar-14
Netherlands|Mar-24
Non-russian sequences with later dates (5)
Asia
Europe
Russia/Chechenya-83803/2020|Mar-26
1
E
Sweden|Mar-09
Canada|Mar-07
USA|Mar-04
Belgium|Mar-04
France|Feb-29
Non-russian sequences with later dates (25)
Europe
Australia
Africa
North America
Asia
Russia/Khabarovsk-71403/2020|Mar-19
1
F
England|Mar-29
Switzerland|Apr-06
Iceland|Mar-20
Non-russian sequences with later dates (1)
Asia
Europe
Australia
South America
North America
Russia/Kipen-RII6907V/2020|Apr-19
1
G
India
Russia/Sakhalin-84401/2020|Mar-27
1
H
Denmark|Mar-15
Non-russian sequences with later dates (1)
Denmark
Russia/Chelyabinsk-85311/2020|Mar-28
1
I
Scotland|Mar-04
Lithuania|Feb-28
Belgium|Mar-05
Sweden|Mar-10
Italy|Mar-03
Finland|Mar-04
Non-russian sequences with later dates (32)
Asia
North America
Russia
Europe
Australia
Russia/Moscow-62501/2020|Mar-11
1
J
North America|Mar-10
Europe|Feb-24
Non-russian sequences with later dates (30)
Asia
Europe
North America
Russia/Moscow-80112/2020|Mar-23
1
K
Non-russian sequences with later dates (1)
Scotland
England
Russia/SCPM-O-01/2020|Mar-20
1
L
Iceland|Mar-16
Scotland|Mar-12
Non-russian sequences with later dates (1)
Europe
Russia
Russia/SCPM-O-03/2020|Mar-20
1
M
France|Feb-26
Denmark|Mar-10
Non-russian sequences with later dates (13)
Asia
Russia
Algeria
Fujian
North America
NanChang
Europe
Africa
South America
Australia
Russia/StPetersburg-3524/2020|Mar-15
1
N
Germany|Mar-11
Denmark|Mar-13
England|Mar-14
Netherlands|Mar-01
Slovakia|Mar-08
USA|Mar-07
Non-russian sequences with later dates (6)
North America
Europe
Russia/StPetersburg-73603/2020|Mar-20
1
O
Australia|Mar-18
USA|Mar-18
Jordan|Mar-16
Greece|Mar-10
England|Mar-20
Non-russian sequences with later dates (5)
North America
Australia
Asia
Europe
Russia/StPetersburg-82005/2020|Mar-24
1
P
Denmark|Mar-03
Non-russina sequences with later dates (4)
North America
Europe
South America
Australia
Asia
Russia/SCPM-O-06/2020|Mar-18
1
Q
Non-russian sequences with later dates (1)
Russia/Moscow-62505/2020|Mar-11
1
R
Europe
Australia
Asia
Russia/Moscow-71604/2020|Mar-19
1

Supplementary Figure 3. Russian singletons (continued from Supplementary Figure 2). Each panel (A-R) shows an individual Russian singleton. Notation as in Figs. 5-6.

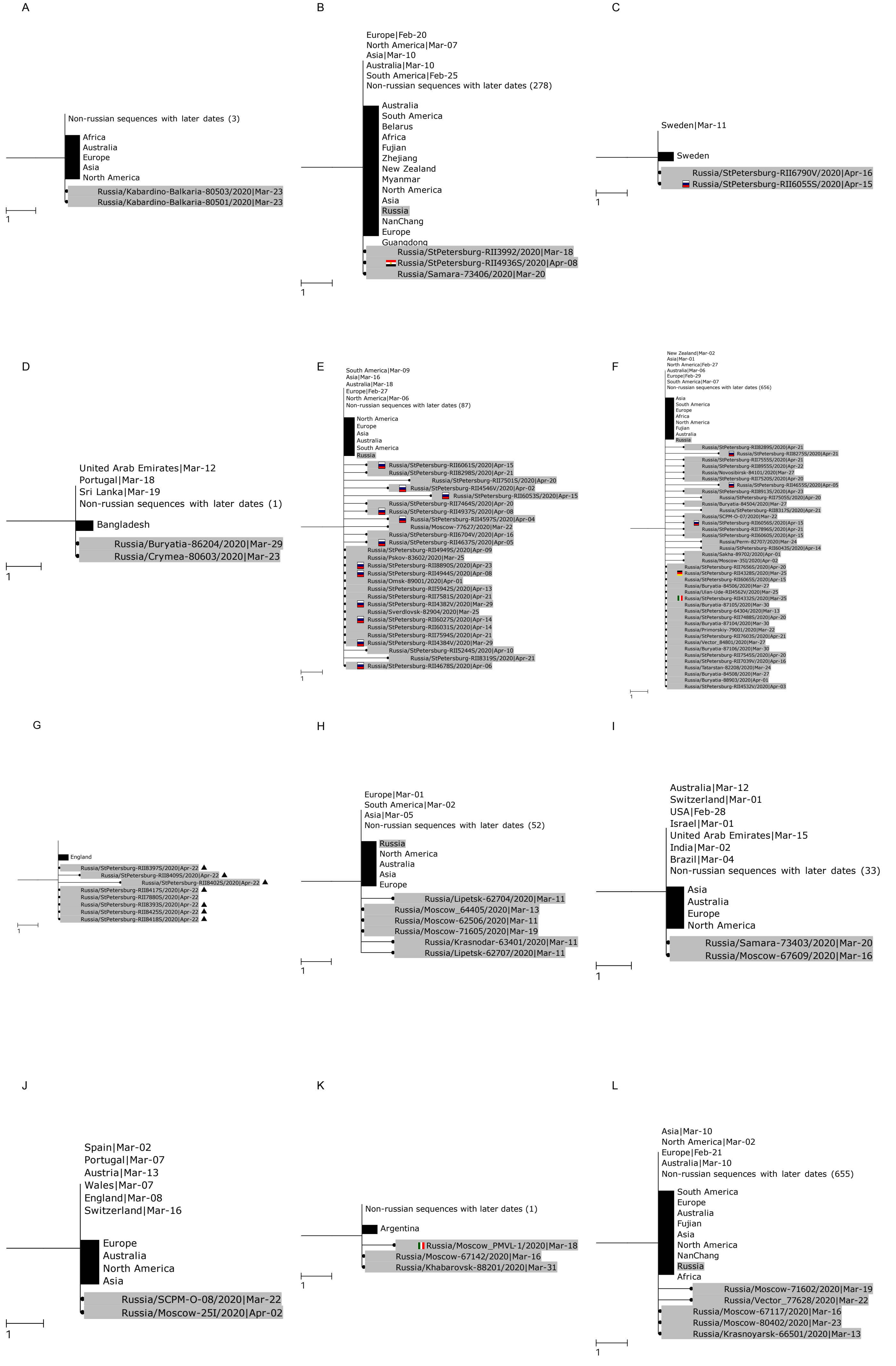
A
Non-russian sequences with later dates (3)
Africa
Australia
Europe
Asia
North America
Russia/Kabardino-Balkaria-80503/2020|Mar-23
Russia/Kabardino-Balkaria-80501/2020|Mar-23
1
B
Europe|Feb-20
North America|Mar-07
Asia|Mar-10
Australia|Mar-10
South America|Feb-25
Non-russian sequences with later dates (278)
Australia
South America
Belarus
Africa
Fujian
Zhejiang
New Zealand
Myanmar
North America
Asia
Russia
NanChang
Europe
Guangdong
Russia/StPetersburg-RII3992/2020|Mar-18
Russia/StPetersburg-RII4936S/2020|Apr-08
Russia/Samara-73406/2020|Mar-20
1
C
Sweden|Mar-11
Sweden
Russia/StPetersburg-RII6790V/2020|Apr-16
Russia/StPetersburg-RII6055S/2020|Apr-15
1
D
United Arab Emirates|Mar-12
Portugal|Mar-18
Sri Lanka|Mar-19
Non-russian sequences with later dates (1)
Bangladesh
Russia/Buryatia-86204/2020|Mar-29
Russia/Crymea-80603/2020|Mar-23
1
E
South America|Mar-09
Asia|Mar-16
Australia|Mar-18
Europe|Feb-27
North America|Mar-06
Non-russian sequences with later dates (87)
North America
Europe
Asia
Australia
South America
Russia
Russia/StPetersburg-RII6061S/2020|Apr-15
Russia/StPetersburg-RII8298S/2020|Apr-21
Russia/StPetersburg-RII7501S/2020|Apr-20
Russia/StPetersburg-RII4546V/2020|Apr-02
Russia/StPetersburg-RII6053S/2020|Apr-15
Russia/StPetersburg-RII7464S/2020|Apr-20
Russia/StPetersburg-RII4937S/2020|Apr-08
Russia/StPetersburg-RII4597S/2020|Apr-04
Russia/Moscow-77627/2020|Mar-22
Russia/StPetersburg-RII6704V/2020|Apr-16
Russia/StPetersburg-RII4637S/2020|Apr-05
Russia/StPetersburg-RII4949S/2020|Apr-09
Russia/Pskov-83602/2020|Mar-25
Russia/StPetersburg-RII8890S/2020|Apr-23
Russia/StPetersburg-RII4944S/2020|Apr-08
Russia/Omsk-89001/2020|Apr-01
Russia/StPetersburg-RII5942S/2020|Apr-13
Russia/StPetersburg-RII7581S/2020|Apr-21
Russia/StPetersburg-RII4382V/2020|Mar-29
Russia/Sverdlovsk-82904/2020|Mar-25
Russia/StPetersburg-RII6027S/2020|Apr-14
Russia/StPetersburg-RII6031S/2020|Apr-14
Russia/StPetersburg-RII7594S/2020|Apr-21
Russia/StPetersburg-RII4384V/2020|Mar-29
Russia/StPetersburg-RII5244S/2020|Apr-10
Russia/StPetersburg-RII8319S/2020|Apr-21
Russia/StPetersburg-RII4678S/2020|Apr-06
1
F
New Zealand|Mar-02
Asia|Mar-01
North America|Feb-27
Australia|Mar-06
Europe|Feb-29
South America|Mar-07
Non-russian sequences with later dates (656)
Asia
South America
Europe
Africa
North America
Fujian
Australia
Russia
Russia/StPetersburg-RII8289S/2020|Apr-21
Russia/StPetersburg-RII8275S/2020|Apr-21
Russia/StPetersburg-RII7555S/2020|Apr-21
Russia/StPetersburg-RII8955S/2020|Apr-22
Russia/Novosibirsk-84101/2020|Mar-27
Russia/StPetersburg-RII7520S/2020|Apr-20
Russia/StPetersburg-RII4655S/2020|Apr-05
Russia/StPetersburg-RII8913S/2020|Apr-23
Russia/StPetersburg-RII7505S/2020|Apr-20
Russia/Buryatia-84504/2020|Mar-27
Russia/StPetersburg-RII8317S/2020|Apr-21
Russia/SCPM-O-07/2020|Mar-22
Russia/StPetersburg-RII6056S/2020|Apr-15
Russia/StPetersburg-RII7896S/2020|Apr-21
Russia/StPetersburg-RII6060S/2020|Apr-15
Russia/Perm-82707/2020|Mar-24
Russia/StPetersburg-RII6043S/2020|Apr-14
Russia/Sakha-89702/2020|Apr-01
Russia/Moscow-35I/2020|Apr-02
Russia/StPetersburg-RII7656S/2020|Apr-20
Russia/StPetersburg-RII4328S/2020|Mar-25
Russia/StPetersburg-RII6065S/2020|Apr-15
Russia/Buryatia-84506/2020|Mar-27
Russia/Ulan-Ude-RII4562V/2020|Mar-25
Russia/StPetersburg-RII4332S/2020|Mar-25
Russia/Buryatia-87105/2020|Mar-30
Russia/StPetersburg-64304/2020|Mar-13
Russia/StPetersburg-RII7488S/2020|Apr-20
Russia/Buryatia-87104/2020|Mar-30
Russia/Primorskiy-79001/2020|Mar-22
Russia/StPetersburg-RII7603S/2020|Apr-21
Russia/Vector_84801/2020|Mar-27
Russia/Buryatia-87106/2020|Mar-30
Russia/StPetersburg-RII7545S/2020|Apr-20
Russia/StPetersburg-RII7039V/2020|Apr-16
Russia/Tatarstan-82208/2020|Mar-24
Russia/Buryatia-84508/2020|Mar-27
Russia/Buryatia-88903/2020|Apr-01
Russia/StPetersburg-RII4532V/2020|Apr-03
1
G
England
Russia/StPetersburg-RII8397S/2020|Apr-22
Russia/StPetersburg-RII8409S/2020|Apr-22
Russia/StPetersburg-RII8402S/2020|Apr-22
Russia/StPetersburg-RII8417S/2020|Apr-22
Russia/StPetersburg-RII7880S/2020|Apr-22
Russia/StPetersburg-RII8393S/2020|Apr-22
Russia/StPetersburg-RII8425S/2020|Apr-22
Russia/StPetersburg-RII8418S/2020|Apr-22
1
H
Europe|Mar-01
South America|Mar-02
Asia|Mar-05
Non-russian sequences with later dates (52)
Russia
North America
Australia
Asia
Europe
Russia/Lipetsk-62704/2020|Mar-11
Russia/Moscow_64405/2020|Mar-13
Russia/Moscow-62506/2020|Mar-11
Russia/Moscow-71605/2020|Mar-19
Russia/Krasnodar-63401/2020|Mar-11
Russia/Lipetsk-62707/2020|Mar-11
1
I
Australia|Mar-12
Switzerland|Mar-01
USA|Feb-28
Israel|Mar-01
United Arab Emirates|Mar-15
India|Mar-02
Brazil|Mar-04
Non-russian sequences with later dates (33)
Asia
Australia
Europe
North America
Russia/Samara-73403/2020|Mar-20
Russia/Moscow-67609/2020|Mar-16
1
J
Spain|Mar-02
Portugal|Mar-07
Austria|Mar-13
Wales|Mar-07
England|Mar-08
Switzerland|Mar-16
Europe
Australia
North America
Asia
Russia/SCPM-O-08/2020|Mar-22
Russia/Moscow-25I/2020|Apr-02
1
K
Non-russian sequences with later dates (1)
Argentina
Russia/Moscow_PMVL-1/2020|Mar-18
Russia/Moscow-67142/2020|Mar-16
Russia/Khabarovsk-88201/2020|Mar-31
1
L
Asia|Mar-10
North America|Mar-02
Europe|Feb-21
Australia|Mar-10
Non-russian sequences with later dates (655)
South America
Europe
Australia
Fujian
Asia
North America
NanChang
Russia
Africa
Russia/Moscow-71602/2020|Mar-19
Russia/Vector_77628/2020|Mar-22
Russia/Moscow-67117/2020|Mar-16
Russia/Moscow-80402/2020|Mar-23
Russia/Krasnoyarsk-66501/2020|Mar-13
1

Supplementary Figure 4. Russian stem clusters and stem-derived singletons. Each panel (A-L) shows an independent stem cluster together with its descendent stem-derived singletons. Notation as in Figs. 5-6. Triangles in (G) represent samples associated with the Vreden hospital outbreak (see Fig. 9).

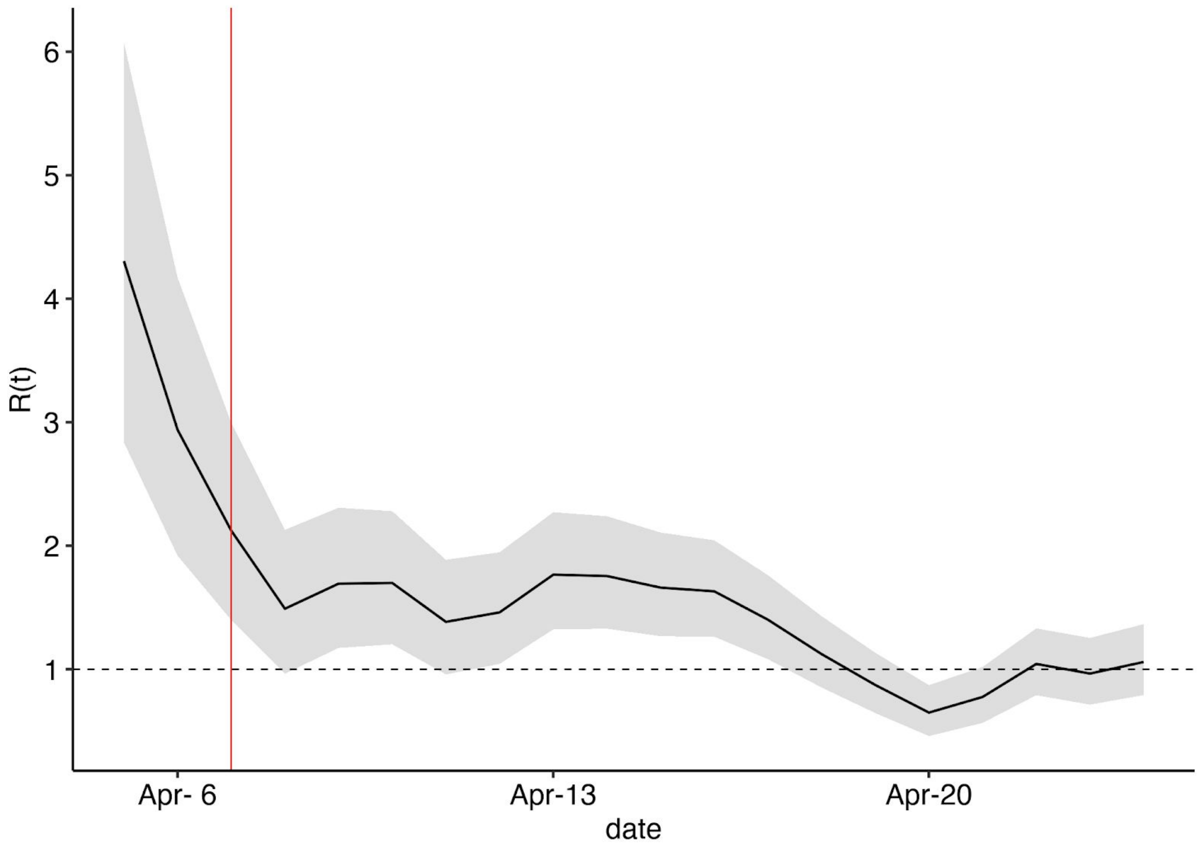

Supplementary Figure 5. Estimated R_e (mean (solid line) and 95% credible interval (shaded area) inferred from incidence data on moderately/severely ill patients in Vreden hospital. The red line marks the introduction of quarantine on Apr-7.

Supplementary Tables

Supplementary Table 1. Estimating the number of introduction events giving rise to Russian stem-derived transmission lineages, Russian stem-derived singletons and Russian stem clusters

	lineages	sequences	travel history		Estimated number of imports
			yes	no	
Russian stem-derived transmission lineages	6	-	2 (50%)	2	=6*0.50=3.00
Russian stem-derived singletons	-	40	1 (14%)	6	=40*0.14=5.60
Russian stem clusters	-	61	4 (36%)	7	=61*0.36=21.96
Total					30.56

Supplementary Table 2. Symptom onset dates for the 11 sequences for which these data are available. Green color is for the sequences collected on April 7; blue, on April 10; and orange, on April 14. Darker colors show sequences for which the symptom onset date differs from the collection date.

Sample id	Symptoms onset date	Collection date

4723	05.04.2020	07.04.2020
4724	05.04.2020	07.04.2020
4726	07.04.2020	07.04.2020
4728	04.04.2020	07.04.2020
4983	10.04.2020	10.04.2020
4984	10.04.2020	10.04.2020
4985	10.04.2020	10.04.2020
4988	09.04.2020	10.04.2020
5643	11.04.2020	14.04.2020
5644	14.04.2020	14.04.2020
5654	13.04.2020	14.04.2020

Supplementary Table 3. Vreden hospital samples per collection date. All the sequences from April 3, 7, 10 and 14 are from group 1. The sequences from April 22 belong to different groups, in particular: 3 sequences from group 1, 7 sequences from group 2 and 4 sequences from group 3.

Date	April 3	April 7	April 10	April 14	April 22
Collection dates	3	17	11	7	14

Multi-rho birth-death skyline model. Supplementary Tables 4, 5, 6 contain the Bayesian estimates of the model parameters for the three datasets comprising groups 1, 2 and 3 (Table 4), groups 1 and 2 (Table 5) and group 1 (Table 6). The estimates of effective reproductive numbers and sampling proportions are consistent throughout all the runs. The tree height corresponds to the dating of the root. Group 1 is suspected to correspond to the first introduction event, so its root corresponds to the suspected beginning of the outbreak. The

dating of the root for the two other datasets provide evidence for multiple introductions. We used Tracer v1.7.1 [1] to summarise the results.

Supplementary Table 4. Phylodynamic parameter estimates for groups 1, 2 and 3. The parameter estimates obtained using BEAST2 with the multi-rho birth-death skyline model.

Parameter	Estimate	95% credible interval
TMRCA date	February 21	January 20 - March 21
clockRate	9.41E-4	[8.44E-4, 1.04E-3]
rho1	0.19	[0.03, 0.39]
rho2	0.44	[0.25, 0.64]
rho3	0.43	[0.21, 0.66]
rho4	0.37	[0.13, 0.63]
rho5	0.61	[0.21, 1.00]
reproductiveNumber1<br>*before March 27*	0.94	[0.54, 1.41]
reproductiveNumber2<br>*March 27-April 8*	3.00	[1.85, 4.25]
reproductiveNumber3<br>*after April 8*	1.76	[0.91, 2.71]

Supplementary Table 5. Phylodynamic parameter estimates for groups 1 and 2. The parameter estimates obtained using BEAST2 with the multi-rho birth-death skyline model.

Parameter	Estimate	95% credible interval
TMRCA date	March 24	March 6 - April 1
clockRate	9.40E-4	[8.44E-4, 1.04E-3]
rho1	0.26	[0.05, 0.51]
rho2	0.48	[0.28, 0.69]

rho3	0.50	[0.25, 0.74]
rho4	0.48	[0.19, 0.77]
rho5	0.65	[0.22, 1.00]
reproductiveNumber1 *before March 27*	0.67	[0.08, 1.01]
reproductiveNumber2 *March 27-April 8*	3.70	[2.08, 5.48]
reproductiveNumber3 *after April 8*	1.70	[0.68, 2.84]

Supplementary Table 6. Phylodynamic parameter estimates for group 1. The parameter estimates obtained using BEAST2 with the multi-rho birth-death skyline model.

Parameter	Estimate	95% credible interval
TMRCA date	March 26	March 13 - April 2
clockRate	9.36E-4	[8.40E-4, 1.03E-3]
rho1	0.28	[0.05, 0.56]
rho2	0.52	[0.30, 0.74]
rho3	0.56	[0.30, 0.82]
rho4	0.56	[0.22, 0.88]
rho5	0.45	[0.03, 0.94]
reproductiveNumber1 *before March 27*	1.21	[0.40, 2.95]
reproductiveNumber2 *March 27-April 8*	3.64	[2.01, 5.43]
reproductiveNumber3 *after April 8*	1.85	[0.77, 3.06]

Multi-rho birth-death skyline model with independent tree models. We ran the multi-rho birth-death skyline model on groups 1, 2 and 3 assuming that each group has an independent

tree model in order to address possible biases due to the population structure given the strong evidence of three independent introductions into the hospital similar to the analysis in [2] (Supplementary Table 7). Though, groups 2 and 3 have only a few sequences representing each of them collected on a single date of April 22. We find parameter estimates for groups 2 and 3 seem to be misleading in this model.

Supplementary Table 7. Phylodynamic parameter estimates for groups 1, 2 and 3 with independent tree models. The parameter estimates obtained using BEAST2 with the multi-rho birth-death skyline model with three separate trees.

Parameter	Estimate	95% credible interval
TMRCA date 1	March 26	March 19 - April 2
TMRCA date 2	April 3	March 15 - April 17
TMRCA date 3	March 27	March 2 - April 15
clockRate	9.33E-4	[8.36E-4, 1.03E-3]
rho1.1	0.27	[0.04, 0.55]
rho1.2	0.53	[0.32, 0.76]
rho1.3	0.57	[0.29, 0.81]
rho1.4	0.57	[0.24, 0.89]
rho1.5	0.45	[0.01, 0.92]
rho2	0.54	[0.11, 1.00]
rho3	0.43	[7.82E-3, 0.92]
reproductiveNumber1.1 *before April 8*	3.44	[1.73, 5.48]
reproductiveNumber1.2 *after April 8*	1.89	[0.80, 3.13]
reproductiveNumber2.1 *before April 8*	1.15	[0.41, 2.65]
reproductiveNumber2.2 *after April 8*	2.13	[0.88, 3.63]
reproductiveNumber3.1	1.11	[0.45, 2.42]

before April 8		
reproductiveNumber3.2<br>*after April 8*	1.76	[0.64, 3.09]

Serial birth-death skyline model.

The fifty two Vreden samples were collected on 5 distinct dates, with a substantial lag between subsequent collection dates (see Supplementary Table 3). For some of the samples, sample collection date could differ substantially from the symptoms onset date. We expect that there might be possible bias due to the loss of within-patient variation due to variant calling procedure. To address this possible bias, we used the symptoms onset date instead of the collection date in BEAST2 analysis and the tip-sampling for the rest of the samples. For 11 samples, the symptoms onset dates were known (Supplementary Table 2). For each of the remaining 41 samples, we produced a posterior estimate of its symptoms onset date by using a uniform prior between March 31st and the collection date. The possible problem of this approach though is the assumption of the birth-death skyline model that sampling immediately results in the death of the lineage. In other words, the resulting model assumes that the patients were isolated immediately after showing the symptoms. We could not verify this assumption, and it is likely that the patients were isolated only after the Covid-19 was confirmed by PCR test. More detailed analysis including simulations are needed to verify whether this approach is more or less reliable than the birth-death model with rho-sampling, which we used in the main text.

Supplementary Tables 8, 9, 10 contain the Bayesian estimates of the model parameters for the three datasets comprising groups 1, 2 and 3 (Table 4), groups 1 and 2 (Table 5) and group 1 (Table 6). The estimates of effective reproductive numbers and sampling proportions are consistent throughout all the runs. The tree height corresponds to the dating of the root. Group 1 is suspected to correspond to the first introduction event, so its root corresponds to the suspected beginning of the outbreak. The dating of the root for the two other datasets provide evidence for multiple introductions. We used Tracer [1] to summarise the results.

Supplementary Table 8. Phylodynamic parameter estimates for groups 1, 2 and 3. The parameter estimates obtained using BEAST2 with the serial birth-death skyline model.

Parameter	Estimate	95% credible interval
TMRCA date	February 4	January 1 - March 7
reproductiveNumber1<br>*before March 27*	0.92	[0.60, 1.16]
reproductiveNumber2<br>*March 27-April 8*	3.72	[2.48, 5.05]

reproductiveNumber3<br>*after April 8*	1.38	[0.48, 2.41]
samplingProportion1	0.0 (fixed)	--
samplingProportion2	0.79	[0.46, 1.00]
samplingProportion3	0.10	[0.01, 0.25]
samplingProportion4	0.01	[4.77E-8, 0.05]
clockRate	9.43E-4	[8.46E-4; 1.04E-3]

Supplementary Table 9. Phylodynamic parameter estimates for groups 1 and 2. The parameter estimates obtained using BEAST2 with the serial birth-death skyline model.

Parameter	Estimate	95% credible interval
TMRCA date	March 15	February 25 - March 31
reproductiveNumber1<br>*before March 27*	1.12	[0.46, 2.42]
reproductiveNumber2<br>*March 27-April 8*	3.96	[2.52, 5.49]
reproductiveNumber3<br>*after April 8*	1.30	[0.47, 2.26]
samplingProportion1	0.0 (fixed)	--
samplingProportion2	0.81	[0.50, 1.00]
samplingProportion3	0.15	[0.02, 0.35]
samplingProportion4	0.02	[1.40E-7, 0.06]
clockRate	9.40E-4	[8.44E-4; 1.04E-3]

Supplementary Table 10. Phylodynamic parameter estimates for group 1. The parameter estimates obtained using BEAST2 with the serial birth-death skyline model.

Parameter	Estimate	95% credible interval
TMRCA date	March 23	March 11 - March 30
reproductiveNumber1<br>*before March 27*	1.28	[0.42, 3.12]
reproductiveNumber2<br>*March 27-April 8*	4.02	[2.46, 5.69]
reproductiveNumber3<br>*after April 8*	1.30	[0.48, 2.26]
samplingProportion1	0.0 (fixed)	--
samplingProportion2	0.82	[0.52, 1.00]
samplingProportion3	0.15	[0.02, 0.35]
samplingProportion4	0.02	[3.35E-8, 0.05]
clockRate	9.38E-4	[8.42E-4; 1.03E-3]

Supplementary Table 11. Priors used in the analysis under the birth-death skyline model. The clockRate prior was used according to the posterior estimates from the UK analysis [3]. Other priors are the same or similar to those used in the birth-death skyline analysis in [4].

Model	Parameter	Prior distribution
HKY	Gamma shape	Exponential(0.5)
	Kappa	Log Normal(1.0, 1.25)
Strict clock	Clock rate<br>(per bp per year)	Normal(9.41e-4, 4.99e-5)
Birth Death Skyline	Effective reproductive number	Log Normal(0.8, 0.5)

	Date of infection origin	Uniform(0, 1000)
	Become uninfectious rate	36.5 (fixed)
multi-rho	Rho sampling probability	Beta(1, 1)
serial	Sampling proportion	Uniform(0, 1)

Supplementary References

1. Rambaut, A., Drummond, A. J., Xie, D., Baele, G. & Suchard, M. A. Posterior Summarization in Bayesian Phylogenetics Using Tracer 1.7. *Systematic Biology* vol. 67 901–904 (2018).
2. Vasylyeva, T. I. *et al.* Tracing the Impact of Public Health Interventions on HIV-1 Transmission in Portugal Using Molecular Epidemiology. *J. Infect. Dis.* **220**, 233–243 (2019).
3. Pybus, O. *et al.* Preliminary analysis of SARS-CoV-2 importation & establishment of UK transmission lineages. *Virological* https://virological.org/t/preliminary-analysis-of-sars-cov-2-importation-establishment-of-uk-transmission-lineages/507/ (2020).
4. Stadler, T. Phylodynamic Analyses of outbreaks in China, Italy, Washington State (USA), and the Diamond Princess. *Virological* https://virological.org/t/phylodynamic-analyses-of-outbreaks-in-china-italy-washington-state-usa-and-the-diamond-princess/439 (2020).
