# Supplementary material for: Genomic epidemiology of the early stages of the SARS-CoV-2 outbreak in Russia
Source: Nat Commun. 2021 Jan 28;12:649. doi: 10.1038/s41467-020-20880-z (PMC7844267; doi:10.1038/s41467-020-20880-z)
Supplement: Supplementary file 3 — Descriptions of Additional Supplementary Files [file 41467_2020_20880_MOESM3_ESM.pdf]

Supplementary Data 1. Sample preparation details including primer sets used, PCR temperature profiles, barcode sequences and .fastq filenames uploaded to SRA, available as a separate file Supplementary_Data_1.xlsx.

Supplementary Data 2. Acknowledgement table containing GISAID sequences used in the analysis, available as a separate file Supplementary_Data_2.xlsx.

Supplementary Data 3. List of sequences excluded from the final dataset, available as a separate file Supplementary_Data_3.xlsx.

Supplementary Data 4. List of Russian SARS-CoV-2 genomes used in the analysis including samples sequenced in the study (‘Sequenced in this study’ column) and travel history (‘Travel history’ column), available as a separate file Supplementary_Data_4.xlsx.
