# Supplementary material for: Genomic epidemiology of the early stages of the SARS-CoV-2 outbreak in Russia
Source: Nat Commun. 2021 Jan 28;12:649. doi: 10.1038/s41467-020-20880-z (PMC7844267; doi:10.1038/s41467-020-20880-z)
Supplement: Supplementary file 8 — Reporting summary [file 41467_2020_20880_MOESM8_ESM.pdf]

nature research

Corresponding author(s): Georgii Bazykin
Last updated by author(s): Nov 30, 2020

Reporting Summary

Nature Research wishes to improve the reproducibility of the work that we publish. This form provides structure for consistency and transparency in reporting. For further information on Nature Research policies, see our Editorial Policies and the Editorial Policy Checklist.

Statistics

For all statistical analyses, confirm that the following items are present in the figure legend, table legend, main text, or Methods section.

n/a	Confirmed	
☐	☒	The exact sample size (*n*) for each experimental group/condition, given as a discrete number and unit of measurement
☐	☒	A statement on whether measurements were taken from distinct samples or whether the same sample was measured repeatedly
☐	☒	The statistical test(s) used AND whether they are one- or two-sided<br>*Only common tests should be described solely by name; describe more complex techniques in the Methods section.*
☐	☒	A description of all covariates tested
☐	☒	A description of any assumptions or corrections, such as tests of normality and adjustment for multiple comparisons
☐	☒	A full description of the statistical parameters including central tendency (e.g. means) or other basic estimates (e.g. regression coefficient) AND variation (e.g. standard deviation) or associated estimates of uncertainty (e.g. confidence intervals)
☐	☒	For null hypothesis testing, the test statistic (e.g. *F*, *t*, *r*) with confidence intervals, effect sizes, degrees of freedom and *P* value noted<br>*Give P values as exact values whenever suitable.*
☐	☒	For Bayesian analysis, information on the choice of priors and Markov chain Monte Carlo settings
☒	☐	For hierarchical and complex designs, identification of the appropriate level for tests and full reporting of outcomes
☐	☒	Estimates of effect sizes (e.g. Cohen's *d*, Pearson's *r*), indicating how they were calculated

Our web collection on statistics for biologists contains articles on many of the points above.

Software and code

Policy information about availability of computer code

Data collection	minION ONT
Data analysis	minION data: guppy_basecaller (v3.6.0), Porechop (v0.2.4), minimap2 (v2.17), SAMtools-mpileup (v1.10), sam2tsv (v20200206), IGV (v2.8.0); dataset preparation: MAFFT (v7.453), IQ-Tree (v1.6.12), TreeTime (v0.7.5), R 3.6.3, Python 3.6; phylodynamics: BEAST2 (v2.6.2), Tracer (v1.7.1), EpiEstim R package (v2.2-3); visualization: ETE3 Python3 toolkit (v2.3.2), iTOL (v4), ggplot R package (v3.3.0), FigTree (v1.4.4). Custom scripts used for data visualization are available at https://github.com/garushyants/covid_russia_early.

For manuscripts utilizing custom algorithms or software that are central to the research but not yet described in published literature, software must be made available to editors and reviewers. We strongly encourage code deposition in a community repository (e.g. GitHub). See the Nature Research guidelines for submitting code & software for further information.

Data

Policy information about availability of data

All manuscripts must include a data availability statement. This statement should provide the following information, where applicable:

- Accession codes, unique identifiers, or web links for publicly available datasets
- A list of figures that have associated raw data
- A description of any restrictions on data availability

GISAID accession IDs of SARS-CoV-2 consensus sequences produced in this study are provided in Supplementary Data 5. Data that support the findings of this study have been deposited at the SRA under accession numbers SRX8723172-SRX8723344, BioProject PRJNA645970. Data referenced in this study is available in Genbank under accession code MN908947.3.

Field-specific reporting

Please select the one below that is the best fit for your research. If you are not sure, read the appropriate sections before making your selection.

- [x] Life sciences
- [ ] Behavioural & social sciences
- [ ] Ecological, evolutionary & environmental sciences

For a reference copy of the document with all sections, see nature.com/documents/nr-reporting-summary-flat.pdf

Life sciences study design

All studies must disclose on these points even when the disclosure is negative.

Sample size	Sample sizes were not predetermined prior to the study. For sequencing, we used all obtained samples with sufficient Ct values (below 30), for a total of 202 out of the 574 samples obtained at the National Influenza Centre in Saint Petersburg, Russia by 2020-04-22. Of 202 samples, 136 were successfully sequenced. These were combined with all SARS-CoV-2 sequence data with Russian location available in GISAID on 2020-05-26.
Data exclusions	One sample produced in this study (hCoV-19/Russia/Ulan-Ude-RII4560S/2020) was excluded from the analysis due to a poor quality of its consensus sequence. Exclusion criteria were pre-established and shared between the data downloaded from GISAID and the data generated in this study.
Replication	N/A as this study didn't produce any experimental findings.
Randomization	N/A as no experimental groups were defined in this study.
Blinding	N/A as no group allocation was performed.

Reporting for specific materials, systems and methods

We require information from authors about some types of materials, experimental systems and methods used in many studies. Here, indicate whether each material, system or method listed is relevant to your study. If you are not sure if a list item applies to your research, read the appropriate section before selecting a response.

Materials & experimental systems

n/a	Involved in the study	
x		Antibodies
	x	Eukaryotic cell lines
x		Palaeontology and archaeology
x		Animals and other organisms
	x	Human research participants
x		Clinical data
x		Dual use research of concern

Methods

n/a	Involved in the study	
x		ChIP-seq
x		Flow cytometry
x		MRI-based neuroimaging

Eukaryotic cell lines

Policy information about cell lines

Cell line source(s)	Vero cell line (ATCC #CCL-81) were purchased from ATCC.
Authentication	The cell line was not authenticated.
Mycoplasma contamination	Negative
Commonly misidentified lines (See ICLAC register)	No commonly misidentified cell lines were used in the study.

Human research participants

Policy information about studies involving human research participants

Population characteristics	The majority of samples were collected in Saint Petersburg (133). Two samples were obtained from Republic of Buryatia and one sample was obtained from Leningrad Region. Overall, 136 samples were sequenced with 47 samples from males (median age 51,5 years, range - 2 month-91) and 81 from females (median age 57 years, range - 18-95). For eight samples, gender information was not available. Out of 136 samples, 52 samples were obtained from patients from the Vreden hospital with 16 samples from males (median age 58 years, range - 24-82) and 34 from females (median age 61 years, range - 32-84). All patients have recovered from the infection.
Recruitment	Samples were obtained within routine and hospital-based surveillance conducted on a regular basis, including samples from the Vreden hospital.
Ethics oversight	Samples used in this study were collected as part of approved ongoing surveillance conducted by the Smorodintsev Research Institute of Influenza. Written informed consent was obtained from all subjects. All samples were de-identified prior to receipt by the study team. The study was presented to the Local Ethics Committee at the Smorodintsev Research Institute of Influenza. The Committee concluded (protocol #151) that the study does not make use of new identifiable biological samples and does not bring forward any new sensitive data. Therefore, according to the rules of the Committee and national regulations this project does not require ethical approval.

Note that full information on the approval of the study protocol must also be provided in the manuscript.
